# Associations of Maternal Stress, Prenatal Exposure to Per- and Polyfluoroalkyl Substances (PFAS), and Demographic Risk Factors with Birth Outcomes and Offspring Neurodevelopment: An Overview of the ECHO.CA.IL Prospective Birth Cohorts

**DOI:** 10.3390/ijerph18020742

**Published:** 2021-01-16

**Authors:** Stephanie M. Eick, Elizabeth A. Enright, Sarah D. Geiger, Kelsey L. C. Dzwilewski, Erin DeMicco, Sabrina Smith, June-Soo Park, Andrea Aguiar, Tracey J. Woodruff, Rachel Morello-Frosch, Susan L. Schantz

**Affiliations:** 1 Program on Reproductive Health and the Environment, Department of Obstetrics, Gynecology and Reproductive Sciences, University of California, San Francisco, CA 94143, USA; erin.demicco@ucsf.edu (E.D.); tracey.woodruff@ucsf.edu (T.J.W.); rmf@berkeley.edu (R.M.-F.); 2 Beckman Institute for Advanced Science and Technology, University of Illinois at Urbana-Champaign, Champaign, IL 61801, USA; smurphy7@illinois.edu (S.D.G.); kelsey.dzw@gmail.com (K.L.C.D.); aaguiar@illinois.edu (A.A.); schantz@illinois.edu (S.L.S.); 3 Department of Psychology, University of Illinois at Urbana-Champaign, Champaign, IL 61820, USA; 4 Department of Kinesiology and Community Health, University of Illinois at Urbana-Champaign, Champaign, IL 61820, USA; 5 Environmental Chemistry Laboratory, Department of Toxic Substances Control, California Environmental Protection Agency, Berkeley, CA 94710, USA; sabrina.smith@dtsc.ca.gov (S.S.); june-soo.park@dtsc.ca.gov (J.-S.P.); 6 Department of Comparative Biosciences, University of Illinois at Urbana-Champaign, Champaign, IL 61802, USA; 7 Department of Environmental Science, Policy and Management and School of Public Health, University of California, Berkeley, CA 94720, USA

**Keywords:** psychosocial stress, health disparities, birth outcomes, neurodevelopment, per- and polyfluoroalkyl substances

## Abstract

Background. Infants whose mothers experience greater psychosocial stress and environmental chemical exposures during pregnancy may face greater rates of preterm birth, lower birth weight, and impaired neurodevelopment. Methods. ECHO.CA.IL is composed of two cohorts, Chemicals in Our Bodies (CIOB; *n* = 822 pregnant women and *n* = 286 infants) and Illinois Kids Development Study (IKIDS; *n* = 565 mother-infant pairs), which recruit pregnant women from San Francisco, CA and Urbana-Champaign, IL, respectively. We examined associations between demographic characteristics and gestational age, birth weight z-scores, and cognition at 7.5 months across these two cohorts using linear models. We also examined differences in biomarkers of exposure to per- and polyfluoroalkyl substances (PFAS), measured in second-trimester serum, and psychosocial stressors by cohort and participant demographics. Results. To date, these cohorts have recruited over 1300 pregnant women combined. IKIDS has mothers who are majority white (80%), whereas CIOB mothers are racially and ethnically diverse (38% white, 34% Hispanic, 17% Asian/Pacific Islander). Compared to CIOB, median levels of PFOS, a specific PFAS congener, are higher in IKIDS (2.45 ng/mL versus 1.94 ng/mL), while psychosocial stressors are higher among CIOB. Across both cohorts, women who were non-white and single had lower birth weight z-scores relative to white women and married women, respectively. Demographic characteristics are not associated with cognitive outcomes at 7.5 months. Conclusions. This profile of the ECHO.CA.IL cohort found that mothers and their infants who vary in terms of socioeconomic status, race/ethnicity, and geographic location are similar in many of our measures of exposures and cognitive outcomes. Similar to past work, we found that non-white and single women had lower birth weight infants than white and married women. We also found differences in levels of PFOS and psychosocial stressors based on geographic location.

## 1. Introduction

Human exposure to environmental chemical and non-chemical (i.e., psychosocial) stressors is ubiquitous, and exposures have been linked to adverse outcomes ranging from impaired cognition and brain function [1,2] to fertility problems [3,4]. During the prenatal period, chemical exposures and maternal stress may be especially detrimental for developmental outcomes in offspring [5,6]. Previous studies have found that women who experience certain psychosocial stressors, including increased perceived stress, depression, and discrimination, are at higher risk of adverse birth outcomes, including preterm birth and lower birth weight and have children who experience worse neurocognitive outcomes [7,8,9,10]. Additionally, certain endocrine-disrupting chemicals (EDCs) commonly found in household goods, including per- and polyfluoroalkyl substances (PFAS) and phenols, are detectable in >90% of the U.S. population-based on studies conducted within the National Health and Nutrition Examination Survey (NHANES) [11,12]. These chemicals pass through the placenta and can affect the developing fetus [13,14], leading to adverse birth outcomes [15,16,17]. Prenatal exposure to PFAS and non-chemical stressors also has been linked to impaired cognition in childhood [10,18,19]. However, these associations have been inconsistent across study populations.

Environmental chemicals and psychosocial stressors may be contributing factors to health inequities observed in maternal and child health outcomes across socioeconomic and racial/ethnic groups. For example, Black women are more likely to experience higher levels of discrimination, poorer neighborhood quality, and more stressful life events relative to white women [20]. Furthermore, these stressors are risk factors for prenatal depression [21], which is associated with adverse birth outcomes [22]. A recent meta-analysis also found that stressors can lead to adverse neurodevelopmental outcomes [23]. Similarly, levels of certain PFAS differ across socioeconomic status (SES) groups [24,25]. The cumulative effects and interactions of psychosocial stressors and environmental chemicals are beginning to be studied in more depth [26]. However, one of the challenges in assessing the extent to which certain demographic groups are more vulnerable to the effects of EDCs is the small sample size of many cohorts.

In response, the Environmental influences on Child Health Outcomes (ECHO) program was developed by the National Institutes of Health (NIH). ECHO combines existing longitudinal pregnancy and child cohorts [26]. In response to the ECHO initiative, two ongoing prospective birth cohorts, Chemicals in Our Bodies (CIOB) and Illinois Kids Development Study (IKIDS), integrated to form ECHO.CA.IL, a geographically, socio-economically, racially and ethnically diverse cohort. ECHO.CA.IL aims to evaluate the relationship between prenatal exposure to EDCs, prenatal stress, and measures of adverse birth outcomes and cognitive development of offspring in infancy and early childhood. Additionally, ECHO.CA.IL will evaluate if maternal stress modifies the observed relationships between EDCs and adverse birth outcomes or cognitive development. To date, epidemiologic studies examining cognitive development have primarily focused on childhood assessments [2], as there are limited standardized measures to test infant cognition. In an advancement over prior work, ECHO.CA.IL uses novel methods more typically used in developmental psychology, including eye-tracking, to assess multiple indices of infant cognition [27]. These measures are sensitive to early life chemical and stress exposures and allow us to assess whether there are changes in infants’ visual attention, information processing speed, and/or recognition memory in response to chemical and non-chemical stressors [27,28].

To date, the ECHO.CA.IL cohorts have recruited over 1300 pregnant women. Here, we provide an overview of ECHO.CA.IL, including participant demographic characteristics, and examine associations between demographic variables, birth outcomes, and infant cognition at 7.5 months across the cohorts. We also assess differences in PFAS and psychosocial stress levels in relation to demographic characteristics.

## 2. Methods

### 2.1. Study Population

The CIOB and IKIDS longitudinal cohorts were separately launched in San Francisco, CA in 2014 and Champaign-Urbana, IL in 2013, respectively, as part of the NIEHS/USEPA Children’s Environmental Health Centers program. In 2016, the two cohorts merged to form ECHO.CA.IL with an overall goal to examine the individual and cumulative effects of prenatal psychosocial stressors and EDC exposures on birth outcomes and cognitive development during infancy (7–8 months) and early childhood (2 and 4 years old). In the context of this study, EDC exposure indicates individual biomarkers of exposure and may reflect the larger body burdens of chemicals to which an individual is exposed. ECHO.CA.IL plans to recruit 1490 pregnant women (720 in IKIDS and 770 in CIOB) and follow at least 1100 infants (500 in IKIDS and 600 in CIOB) through age 4. Maternal psychosocial stressors and chemical biomarkers of exposure are measured during pregnancy, and there are multiple visits during infancy and childhood (7.5 months, 2, 3, and 4 years in both cohorts) to gain a comprehensive picture of development. ECHO.CA.IL specifically focuses on measuring self-reported prenatal psychosocial stressor exposures as well as biomarkers of the chronic stress response. These biomarkers include maternal-corticotropin releasing hormone (CRH) measured in plasma during the second trimester and telomere length measured in maternal-whole blood during pregnancy, and newborn cord blood collected at delivery. Stress has been linked to changes in biomarkers of stress (ex. CRH and telomere length) [29,30], adverse birth outcomes [22] and neurodevelopment [28]. ECHO.CA.IL also measures concentrations of 12 PFAS in maternal serum and 12 phenols in maternal urine during pregnancy. Both cohorts employ multiple neurocognitive measures when infants are 7.5 months old, including eye-tracking to investigate memory, attention, and information processing speed and the ages and stages questionnaire (ASQ) to assess communication, problem-solving, motor skills, and personal/social skills [31]. In addition, both cohorts collect biospecimens including child hair and urine at 4 years of age and toenail samples at 7.5 months, 2, and 4 years of age, additional measures of neurocognitive development including the child behavior checklist (CBCL) and ASQ at 2 and 4 years of age, and direct measures of language development and cognition (including tasks to measure executive function, inhibitory control, working memory, and numerical understanding) at 4 years of age. As part of ECHO.CA.IL, study staff was blinded to all participant measures of psychosocial stress, environmental chemicals, and neurodevelopment. Medical information and lifestyle questionnaires are also used to answer additional questions about the health and wellbeing of women and their children. In later years, upon completion of ECHO, de-identified datasets containing questionnaires and biological information will be available to the larger scientific community and investigators via the ECHO Data Analysis Center (DAC). The strengthening the reporting of observational studies in epidemiology (STROBE) guidelines [32] were followed in the reporting of this study (Table S1).

#### 2.1.1. Chemicals in Our Bodies Recruitment and Data Collection during Pregnancy

CIOB is a prospective pregnancy cohort and was originally designed to examine the cumulative effects of environmental chemicals and psychosocial stressors on fetal growth [33]. Recruitment for CIOB began in 2014 and is ongoing. Pregnant women are recruited during their second trimester from three University of California, San Francisco hospitals and their children are followed through four years of age. Participants recruited from the Zuckerberg San Francisco General Hospital are primarily lower-income and enrolled in Medi-Cal (California’s Medicaid program), whereas women recruited from Moffitt Long and Mission Bay Hospitals are more economically diverse, and the majority have private health insurance. Eligibility criteria include ≥18 years of age, singleton pregnancy, and English or Spanish speakers. With the exception of gestational hypertension and preeclampsia, women with diagnosed pregnancy complications are ineligible for participation. As part of the study, women complete an interview questionnaire during the 2nd trimester, assessing psychosocial stress, health, and lifestyle of the mother, including product use, tobacco smoke, diet and sleep. Blood samples are obtained from participants during the 2nd trimester, and two spot urine samples are obtained at second and third-trimester prenatal visits. Mothers also consent for study staff to access their medical records, which are linked to the child’s medical record after birth. The Institutional Review Boards (IRB) at the University of California, San Francisco (10-00861) and Berkeley (2010-05-04) approved CIOB, and all participants provide written, informed consent prior to participating.

#### 2.1.2. Illinois Kids Development Study Recruitment and Data Collection during Pregnancy

IKIDS is a prospective pregnancy cohort that is designed to examine how environmental factors, such as stress and exposure to chemicals, during pregnancy impact early neurocognitive development. Recruitment for IKIDS began in 2013 and is ongoing. Infants enrolled in the study before August of 2020 are included in this paper (*n* = 565 infant/mother dyads). Pregnant women are asked if they are interested in learning more about the study when they attend their first prenatal appointment at prenatal clinics located in Champaign and Urbana, IL. Interested mothers receive a phone call from the study staff who describe the study and determine eligibility. Women who plan to give birth at Order of Saint Francis Heart of Mary Medical Center or Carle Foundation Hospital are eligible for the study. Women are eligible if they have not yet reached 15 weeks gestation, are between 18 and 40 years of age, are not pregnant with multiples, speak English as their primary language, have a low-risk pregnancy, and are not already enrolled in the study with another child. Since the study includes multiple follow-up visits throughout pregnancy and with the children after birth, women who do not reside within a 30-min drive of Champaign-Urbana, IL at the time of recruitment are not eligible. Participants are first interviewed and complete questionnaires between 10 and 14 weeks of gestation to assess psychosocial stress, lifestyle, health, and diet, and these interviews and questionnaires were used for the analyses reported here. Additional interviews and questionnaires take place throughout pregnancy. Blood samples are obtained from participants between 16 and 18 weeks of gestation, and first-morning urine samples are collected five times throughout pregnancy at 10–14 weeks, 16–18 weeks, 22–24 weeks, 28–30 weeks, and 34–36 weeks. Within 48 h of birth, the study staff completes an interview at the hospital to gather birth outcome information as well as to complete physical measurements of the newborn baby. Mothers sign the Health Insurance Portability and Accountability Act (HIPPA) forms, allowing researchers to access medical records for the pregnancy and birth. The IRB at the University of Illinois (09498) approved IKIDS, and all participants provide written, informed consent prior to their participation.

### 2.2. Demographics, Lifestyle, and Health Information

Both cohorts collect demographic, lifestyle and health data, including maternal education, marital status, maternal age, race/ethnicity, parity, infant sex, and health information, including pre-pregnancy body mass index (BMI; kg/m^2^). CIOB collects information on maternal education, marital status, maternal age, race/ethnicity, infant sex, and smoking status from an interview questionnaire. If this information is missed on the questionnaire, CIOB abstracts the information from the medical record when available. Information on pre-pregnancy BMI and parity is obtained solely from medical records in CIOB. IKIDS also uses interviews to collect self-reported demographic, health, and lifestyle data, such as maternal education, marital status, maternal age, race/ethnicity, parity, infant sex, smoking status, and BMI (calculated from self-reported pre-pregnancy weight and maternal height).

### 2.3. Per- and Polyfluoroalkyl Substances (PFAS) Measurement

Serum samples from both cohorts are centrifuged and aliquoted prior to storage at −80 °C at cohort sites and later stored at −20 °C at the Environmental Chemical Laboratory at the California Department of Toxic Substances Control (DTSC), which quantified 12 PFAS in both cohorts (perfluoro butane sulfonate (PFBS), perfluorohexanesulphonic acid (PFHxS), perflucorooctane sulfonic acid (PFOS), perfluoroheptanoic acid (PFHpA), perfluorooctanoic acid (PFOA), perfluorononanoic acid (PFNA), perfluorodecanoic acid (PFDeA), perfluoroundecanoic acid (PFUdA), perfluorododecanoic acid (PFDoA), perfluorooctane sulfonamide (PFOSA), methyl-perfluorooctane sulfonamide acetic acid (Me-PFOSA-AcOH), ethyl-perfluorooctane sulfonamide acetic acid (Et-PFOSA-AcOH)). The samples are extracted and analyzed using a Symbiosis Pharma automated online solid-phase extraction system (Spark Holland) coupled with liquid chromatography and tandem mass spectrometry to quantify PFAS. The method detection limit (MDL) is calculated as 3 times the standard deviation of the blank concentrations for all PFAS [33]. Values below the MDL are assigned the machine read value if a signal is detected. Those below the MDL where no signal is obtained are coded as missing. Additional information regarding PFAS measurement is provided elsewhere [33,34].

### 2.4. Phenolic Compounds

Analysis for 12 urinary phenolic compounds (Bisphenol A [BPA], Bisphenol F [BPF], Bisphenol S[BPS], butyl, ethyl, methyl and propyl parabens, triclosan, benzophenone-3 and 2,4- and 2,5-dichlorophenol) is not yet complete. Phenolic compounds will be measured in maternal urine samples by online solid-phase extraction-high-performance liquid chromatography-isotope dilution tandem mass spectrometry-based on methods used in previous research [35,36]. The analysis is currently being done at the Centers for Disease Control and Prevention (CDC) Division of Laboratory Sciences.

### 2.5. Psychosocial Stress and Depression

The perceived stress scale-4 (PSS-4) and PSS-10 [37] are used to measure perceived stress in CIOB and IKIDS, respectively. Perceived stress was harmonized by converting the PSS-4 and PSS-10 to T-scores using the NIH toolbox [38]. T-scores are normed to the adult U.S. population, and higher T-scores correspond to higher stress levels.

Depression was assessed using the Center for Epidemiologic Studies-Depression (CES-D) scale in CIOB [39] and the Edinburg Postnatal Depression Scale (EPDS) [40] in IKIDS. Existing clinical cut points for the CES-D (≥16) [41] and EPDS (≥13) [42] are used to create binary indicators of clinical levels of depression and combined across cohorts.

Participants are asked if any of the following stressful life events occurred in the past year in CIOB and during pregnancy in IKIDS: (1) participant or their partner experienced a job loss or unemployment, (2) participant or a close family member has been seriously injured, ill, or hospitalized, (3) participant or their partner experienced serious legal or financial problems, (4) participant experienced divorce, a separation, or serious relationship difficulties with their partner, and (5) someone close to participant passed away. The number of stressful life events (yes/no) was summed in both cohorts to create a continuous measure (range 0–5) [43,44].

### 2.6. Biomarkers of Chronic Stress Response

Both cohorts measure maternal and newborn telomere length in whole blood and cord blood, obtained during the second trimester and at delivery, respectively, using a quantitative polymerase chain reaction assay that determinates the relative ratio of telomere repeat abundance to single-copy gene abundance [45,46]. CRH is also measured in maternal plasma samples collected during the 2nd trimester using enzyme immunoassay kits and standard operating procedures.

### 2.7. Birth Outcomes

Both cohorts collect data on birth outcomes that have implications for later development [47,48,49]. Specifically, this includes gestational age, birth weight, type of delivery (i.e., vaginal birth or cesarean section), and whether the birth is spontaneous. CIOB obtains information on birth outcomes from medical records. If gestational age is missing on the medical record, it is imputed using the date of the last menstrual period and the date of birth if available. IKIDS collects the majority of birth outcome data directly from mothers at the hospital in a birth interview. IKIDS estimates gestational age at birth based on mothers’ ultrasounds and the day the baby was born. Mothers self-report on the delivery type and whether the birth is spontaneous. Birth weight is collected from crib cards at the hospital.

In both cohorts, preterm birth is defined as gestational age less than 37 weeks and categorized into preterm subtypes, including extremely (less than 27 weeks gestation), moderately (between 27 and 32 weeks gestation), and late preterm (between 32 and 37 weeks gestation) [50]. Birth weight z-scores are calculated using a U.S. population-based reference and are sex-specific [51]. Birth weight z-scores, as opposed to raw birth weight, are used in order to account for gestational age at delivery, as birth weight may be confounded by gestational age.

### 2.8. Visual Recognition Memory (VRM) Outcomes at 7.5 Months

To assess cognitive outcomes, between seven to eight months of age, infants came to the lab to complete a visual recognition memory (VRM) task [27] developed by Susan Rose and colleagues [52] and adapted for use with infrared eye-tracking [27]. In this task, infants view images of faces on a large screen, and infants’ eye movements are tracked by an SR Research EyeLink eye tracker. Eye-tracking is conducted in a quiet room where the television screen showing the stimuli is the only point of interest in order to reduce distractions. Before any stimuli are shown, the eye tracker is calibrated for each infant. During familiarization of the VRM task, infants view two identical faces side-by-side until they have accumulated a total of 20 s of looking time to the stimuli to give them a chance to encode and remember the face. VRM test trials directly follow familiarization, where infants see the face they viewed during familiarization and a new face they have not seen before side-by-side. After infants accumulate 1 s of looking at the faces, the faces remain on the screen for an additional 5 s. Two test trials are shown for each pair of faces. About half of the infants saw the new face on the right side of the screen for the first test trial and on the left side in the second test trial, while the other half of infants saw the reverse. Infants complete 5 sets of trials using 5 different pairs of black and white photographs of human faces. Additional details regarding the VRM task are provided elsewhere [27].

Three key dependent variables are obtained from the VRM task to measure different aspects of cognition: novelty preference, average run duration, and time to familiarization. Novelty preference is measured by taking the proportion of time infants spend looking at the novel face compared to the total amount of time they spend looking at both faces during the test trials to measure recognition memory. If infants remember the familiar face, they should spend a greater proportion of time looking at the novel face [53,54]. Average run duration is assessed in order to measure information processing speed, and faster processors tend to have shorter average run duration. This measure is calculated by averaging the number of time infants spend looking at the stimuli before looking away. Lastly, time to familiarization is used to measure attention by looking at the time to reach the familiarization criteria (e.g., time to accumulate 20 s of looking at the faces during each familiarization trial), and more time off-task will lead to a longer trial duration to meet the looking time criteria. For all three outcome variables on the VRM task, measures are averaged across the five sets of trials and treated as continuous measures. Infants who do not complete all five trials were excluded from analyses in this manuscript.

### 2.9. Additional Neurodevelopment Measures

Additional measures of neurocognitive development outcomes are assessed throughout early childhood. At 7.5 months, 2 years, 3 years, and 4 years of age, IKIDS and CIOB mothers complete the ASQ. Additionally, IKIDS and CIOB mothers complete the CBCL [55] when their child is 2 years old and again when their child is 4 years old. Both cohorts also administer computer tasks at age 4, which assess executive function, number understanding, working memory, attention, and inhibitory control, and an eye-tracking task to access children’s Theory of Mind understanding.

### 2.10. Statistical Analysis

Frequencies, counts, means, and standard deviations (SD) were used to describe the ECHO.CA.IL population. We conducted a complete case analysis, and linear regression models were used to examine associations between demographic characteristics (maternal age, pre-pregnancy BMI, race/ethnicity, maternal education, infant sex, parity, smoking status, and marital status) and gestational age, birth weight z-scores, and VRM outcomes. We conducted a sensitivity analysis for term birth weight as it may be more interpretable than z-scores. Associations were stratified by individual cohort when sample sizes permitted. Given the descriptive nature of this analysis and that there was no one exposure-outcome relationship of interest, we present unadjusted associations.

We examined the distribution of 12 PFAS across the overall ECHO.CA.IL cohort, as well as stratified by CIOB and IKIDS. Median PFAS levels were also compared to women of reproductive age (18–40 years) within the nationally representative NHANES study population from 2013–2014 [56]. Primary analyses focused on the 7 PFAS with >75% detection in the ECHO.CA.IL cohort (PFNA, PFOS, PFOA, Me-PFOSA-AcOH, PFHxS, PFDeA, PFUdA). We estimated the median, geometric mean, and geometric SD of these PFAS in relation to demographic characteristics overall and stratified by cohort. *P*-values were estimated using linear regression models, which used natural log-transformed PFAS concentrations as outcome measures due to skewed distributions.

All statistical analyses were conducted in R Version 4.01.

## 3. Results

### 3.1. Overall, Demographic Data

To date, over 1387 women are enrolled in ECHO.CA.IL (*n* = 822 in CIOB and *n* = 565 in IKIDS) (Table 1). The majority of women across cohorts had a college (26%) or graduate degree (37%), with a slightly higher percentage of women in IKIDS having a graduate education (Table 1). The IKIDS cohort was primarily non-Hispanic white (80%), whereas CIOB was more racially and ethnically diverse with a large percentage of Hispanic participants (34%). Maternal parity was higher in IKIDS than CIOB (60% versus 41% of moms had 1+ prior pregnancies). Mothers from the two cohorts were of similar age and had similar pre-pregnancy BMIs.

### 3.2. Stress

Perceived stress T-scores were slightly higher in CIOB than IKIDS (mean 50 versus 45), as was the prevalence of clinical levels of depressive symptoms (6% versus 4%). Stressful life events were also relatively rare, with women, on average, experiencing less than one stressful life event during pregnancy (Table 1). In both cohorts, the most common stressful life event reported was serious illness or injury of the participant or a close family member. Overall, the mean number of stressful life events and perceived stress T-scores were higher among women who were younger, without a college degree, and non-white (Table S2).

### 3.3. Birth Outcomes

The mean gestational age at delivery was 39 weeks in both cohorts, and there were very few preterms (7% in CIOB and 4% in IKIDS) and low birth weight (5% in CIOB and 1% in IKIDS) births (Table 2). Of the preterm births in both cohorts, 60% were spontaneous, and there were 2, 11, and 69 births classified as extremely, moderately, and late preterm, respectively. In the overall cohort, having less than a college education was associated with shorter gestational age compared to those with a college degree (β = −0.44, 95% confidence interval CI = −0.70, −0.19) (Table 3). When stratifying by cohort, this unadjusted association was stronger in CIOB (β = −0.67, 95% CI = −1.04, −0.31) compared to IKIDS (β = −0.06, 95% CI = −0.42, 0.31) (Table S3).

Across all racial and ethnic groups, being non-white was associated with a reduction in birth weight *z*-scores compared to white women (Table 3). Being single was also associated with lower birth weight z-scores relative to married or cohabitating women (β = −0.48, 95% CI = −0.70, −0.26) in unadjusted models. No clear differences were observed between demographics and birth weight z-scores when stratifying by cohort (Table S4). When term birth weight was the outcome of interest, unadjusted associations were similar to what was observed with birth weight z-scores (Table S5).

### 3.4. VRM

The distributions of VRM outcomes were similar across cohorts, although time to familiarization was slightly longer in CIOB (mean 60 versus 52 s) (Table 2). Maternal demographic characteristics were not associated with any of the VRM outcomes examined at 7.5 months (Table 4).

### 3.5. PFAS

**Figure 1:**
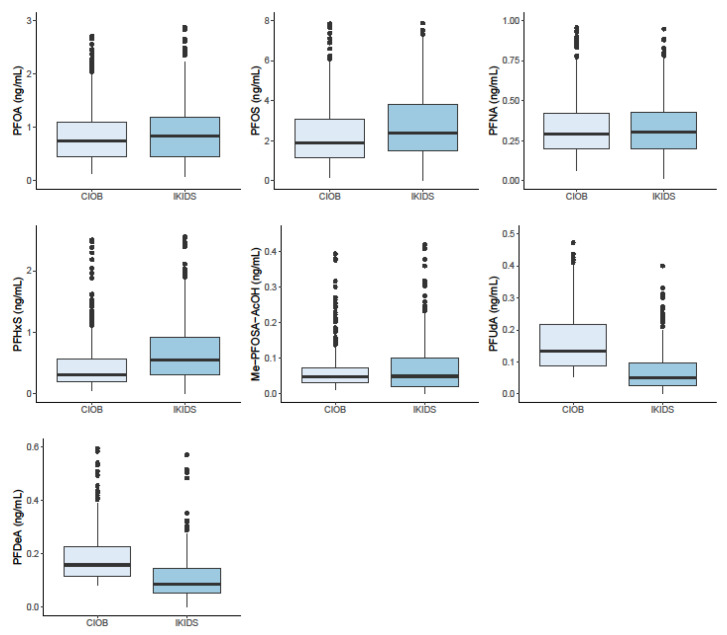
Distribution of polyfluoroalkyl substances (PFAS) (ng/mL) levels with >75% detection stratified by cohort.

**Figure 2:**
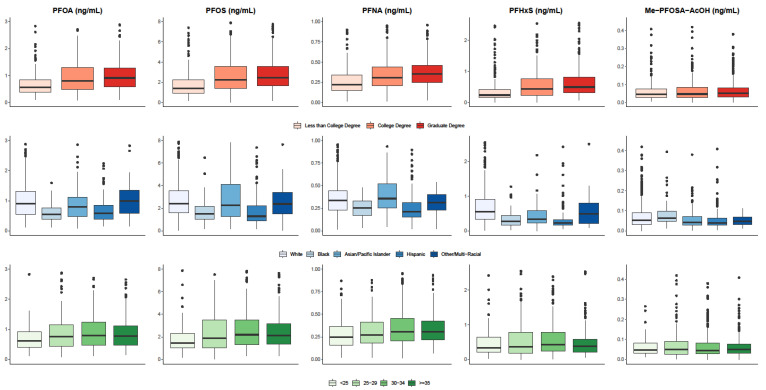
Distribution of PFAS (ng/mL) levels with >95% detection across selected demographic characteristics.

With the exceptions of PFUdA and PFDeA, median levels of PFAS were higher among pregnant women in IKIDS than CIOB (Figure 1; Table S6). PFAS levels in the overall cohort were generally lower than what was observed in reproductive-age women in the NHANES study population (Table S7). Levels of PFOA, PFOS, PFNA, and PFHxS were higher among women with a college or graduate education compared to those with less than a college degree and among women who were married or living with a partner relative to those who were single (Figure 2; Table S8). PFAS levels also varied widely across racial and ethnic groups, with Hispanic and Black women generally having the lowest biomarkers of exposure levels (Figure 2; Table S8).

## 4. Discussion

In 2016, CIOB and IKIDS merged to form ECHO.CA.IL, a demographically diverse cohort spanning two different geographic regions. To date, these cohorts have recruited over 1000 pregnant women combined. Our goal with the present analysis is to describe the study population and discuss how the data are collected and combined in these two pregnancy and child cohorts. We also explored differences in PFAS and psychosocial stress levels and examined associations between demographic characteristics and both neurodevelopment and adverse birth outcomes in this study population.

Comparing cohorts, CIOB and IKIDS were similar on many factors. Mothers were of similar maternal age, had similar pre-pregnancy BMI, and reported low levels of depression and perceived stress. However, the cohorts differed in maternal education with IKIDS mothers having slightly more education, in race/ethnicity where CIOB was more diverse and had a higher percentage of Hispanic and Asian or Pacific Islander mothers, and in a marital status where IKIDS mothers were more likely to be married or cohabitating with their partner than CIOB mothers. Psychosocial stressors were also more prevalent in the CIOB cohort relative to IKIDS. Further, we found that non-white women, younger women, and women without a college degree were more likely to have higher stress levels, which is consistent with past work [57].

In our study, population levels of PFAS were lower overall than what was observed in the NHANES study population during a similar time frame. Most PFAS levels were higher among higher educated women, those married or living with a partner, and women who had one or more prior births. While this is in contrast with prior work that observed higher PFAS levels among pregnant women who are younger, non-white, and with less than a college education [24], these findings are consistent with a prior study showing that increasing household income was associated with higher serum PFAS concentrations during pregnancy [58]. It is possible that higher PFAS levels observed in our cohort may be reflective of behavior and lifestyle factors associated with SES. The varying PFAS levels across demographic characteristics may be attributed to the diversity of our cohort. For example, the IKIDS cohort was somewhat higher educated than CIOB and had higher PFAS levels. Similarly, PFAS levels in our study were lower among Hispanic participants relative to other racial and ethnic groups. This may be driven by the high percentage of Hispanic participants in CIOB that were born outside of the U.S. In the CIOB cohort, foreign-born women had lower PFAS levels compared to those who were not foreign born. This is consistent with prior work in Study of Women’s Health Across the Nation (SWAN) and the Center for the Health Assessment of Mothers and Children of Salinas (CHAMACOS) study indicating that women born outside the U.S. have lower levels of persistent EDCs, including PFAS [59,60].

Future work in the ECHO.CA.IL cohort will examine the individual and cumulative effects of psychosocial stressors and EDCs on birth outcomes and cognitive development during infancy and early childhood. Both cohorts are measuring multiple PFAS and phenols that are not routinely measured during pregnancy and are using a novel infrared eye-tracking method to assess infant and child cognition. Previous work in IKIDS has shown that the VRM task is sensitive to changes in prenatal phthalate levels and others to maternal stress during pregnancy [10,28,61]. In the present analysis, we found that the VRM task was not associated with SES indicators typically associated with cognitive outcomes. It is possible that these social factors do not impact cognition until later in childhood. These cohorts also measure prenatal CRH levels and maternal and newborn telomere length as a biomarker of the stress response and possible physiologic mechanisms linking chemical and stress exposures to adverse health outcomes.

The ECHO.CA.IL study has numerous strengths. Combining two existing cohorts, we enhance the generalizability of our results. The sample size is also larger than many other previous studies examining birth outcomes and developmental effects of stress and chemical exposures during pregnancy. Children in both cohorts are followed through the first four years of life, and neurodevelopment is assessed at numerous time points using a variety of methods. Despite these important strengths, we also acknowledge limitations in the combined cohort. In the present analysis, it was not possible to directly combine the psychosocial stressors across cohorts, as depression was measured using different scales in the two cohorts (EPDS in IKIDS versus CES-D in CIOB). While we did use existing clinical cut points to create binary indicators of depression, misclassification may have occurred. However, this method has been used previously in meta-analyses to combine psychosocial stress scales [62]. When the two cohorts merged to form ECHO.CA.IL in 2016, CIOB began administering the EPDS during pregnancy and at subsequent child visits. To date, this information is not available for analyses; however, future work in this cohort will compare how the EPDS and CES-D compare in CIOB. Questions pertaining to stressful life events were also asked somewhat differently in both cohorts. For example, CIOB asks if someone close to the participant passed away, and IKIDS asks if a close family member has passed away. Further, IKIDS used the PSS-10 to assess perceived stress, whereas CIOB used the PSS-4 to assess perceived stress. Although we used best practices to harmonize data by converting to T-scores [38], past work finds that the same participants will sometimes respond differently to the same question on these two scales [63]. Other methods for the harmonization of psychosocial stressors measures are currently being developed by the ECHO Data Analysis Center (DAC), and future work may harmonize stress measures in this cohort using these methods. Additionally, the ECHO.CA.IL cohort generally has higher education levels than what is observed in the larger U.S. population, which may have implications for generalizability. Lastly, the present analysis may be underpowered to detect differences in VRM outcomes, as a relatively small number of infants in CIOB have completed this task at the time of the present analysis.

## 5. Conclusions

The present study provides an overview of the combined ECHO.CA.IL cohort, a summary of the measures collected, and details about how the data were combined and harmonized. We found that demographic variables were not associated with cognitive development at 7.5 months. In our study, we observed that PFAS and psychosocial stressors vary across these two study populations, observing that PFAS levels were higher among IKIDS participants. This study provides an important baseline, descriptive information that will be considered in future work using the ECHO.CA.IL cohort, examining the cumulative effects of stress and chemical exposures on pregnancy and childhood development.

## Figures and Tables

**Table 1:** Distribution of demographic characteristics and psychosocial stressors in environmental influences on child health outcomes (ECHO).CA.IL cohorts.

Demographics	CIOB (*n* = 822)	IKIDS (*n* = 565)	Total (*n* = 1387)
	*n* (%) or Mean (SD)	*n* (%) or Mean (SD)	*n* (%) or Mean (SD)
Maternal Age (years)			
<25	83 (10%)	48 (8%)	131 (9%)
25–29	106 (13%)	182 (32%)	288 (21%)
30–34	301 (37%)	252 (45%)	553 (40%)
≥35	315 (38%)	83 (15%)	398 (29%)
Missing	17 (2.1%)	0 (0%)	17 (1.2%)
Pre-pregnancy BMI (kg/m^2^)			
Underweight (<18.5 kg/m^2^)	23 (3%)	14 (2%)	37 (3%)
Normal (18.5–24.9 kg/m^2^)	364 (44%)	286 (51%)	650 (47%)
Overweight (25–29.9 kg/m^2^)	164 (20%)	123 (22%)	287 (21%)
Obese (≥30 kg/m^2^)	105 (13%)	138 (24%)	243 (18%)
Missing	166 (20.2%)	4 (0.7%)	170 (12.3%)
Maternal Education			
<College degree	281 (34%)	112 (20%)	393 (28%)
College degree	192 (23%)	197 (35%)	389 (28%)
Graduate degree	290 (35%)	256 (45%)	546 (39%)
Missing	59 (7.2%)	0 (0%)	59 (4.3%)
Maternal Race/Ethnicity			
White	313 (38%)	453 (80%)	766 (55%)
Black	50 (6%)	31 (5%)	81 (6%)
Asian/Pacific Islander	141 (17%)	31 (5%)	172 (12%)
Hispanic	282 (34%)	15 (3%)	297 (21%)
Other/multiracial	25 (3%)	35 (6%)	60 (4%)
Missing	11 (1.3%)	0 (0%)	11 (0.8%)
Infant Sex			
Male	373 (45%)	254 (45%)	627 (45%)
Female	391 (48%)	273 (48%)	664 (48%)
Missing	58 (7.1%)	38 (6.7%)	96 (6.9%)
Parity			
1+ Births	339 (41%)	339 (60%)	678 (49%)
No prior births	366 (45%)	226 (40%)	592 (43%)
Missing	117 (14.2%)	0 (0%)	117 (8.4%)
Smoking Status			
Not current smoker	766 (93%)	556 (98%)	1322 (95%)
Current smoker	10 (1%)	9 (2%)	19 (1%)
Missing	46 (5.6%)	0 (0%)	46 (3.3%)
Marital Status			
Married or living together	639 (78%)	535 (95%)	1174 (85%)
Single	78 (9%)	30 (5%)	108 (8%)
Missing	105 (12.8%)	0 (0%)	105 (7.6%)
Psychosocial Stressors			
Perceived stress (range 0–85)	50 (8.7)	45 (11)	48 (10)
Missing	72 (8.8%)	9 (1.6%)	81 (5.8%)
Clinical Levels of Depression			
No	662 (81%)	529 (94%)	1191 (86%)
Yes	50 (6%)	25 (4%)	75 (5%)
Missing	110 (13.4%)	11 (1.9%)	121 (8.7%)
Stressful life events (range 0–5)	1.0 (1.1)	0.32 (0.66)	0.77 (1.0)
Missing	57 (6.9%)	118 (20.9%)	175 (12.6%)

Note: percentages may not sum to 100 due to rounding, medical record abstraction has not been completed for all CIOB participants, and some data currently noted as missing will be obtained through medical record abstraction. Abbreviations: SD, standard deviation.

**Table 2:** Distribution of birth outcomes and visual recognition memory (VRM) outcomes from face trials in ECHO.CA.IL cohorts.

VRM and Birth Outcomes	CIOB (*n* = 822)	IKIDS (*n* = 565)	Total (*n* = 1387)
	*n* (%) or Mean (SD)	*n* (%) or Mean (SD)	*n* (%) or Mean (SD)
Delivery method			
Vaginal	542 (66%)	361 (64%)	903 (65%)
C-section	135 (16%)	138 (24%)	273 (20%)
Missing	145 (17.6%)	66 (11.7%)	211 (15.2%)
Gestational age (weeks)	39 (2.0)	39 (1.5)	39 (1.8)
Missing	126 (15.3%)	38 (6.7%)	164 (11.8%)
Preterm birth			
Yes	58 (7%)	25 (4%)	83 (6%)
No	638 (78%)	502 (89%)	1140 (82%)
Missing	126 (15.3%)	38 (6.7%)	164 (11.8%)
Birth weight (grams)	3400 (580)	3500 (430)	3400 (530)
Missing	84 (10.2%)	116 (20.5%)	200 (14.4%)
Low birth weight			
Yes	44 (5%)	4 (1%)	48 (3%)
No	694 (84%)	445 (79%)	1139 (82%)
Missing	84 (10.2%)	116 (20.5%)	200 (14.4%)
Birth weight z-score	0.002 (1.0)	0.16 (0.93)	0.063 (0.98)
Missing or incomplete	140 (17.0%)	124 (21.9%)	264 (19.0%)
Novelty preference (proportion) ^1^	56 (6.6)	57 (6.8)	57 (6.8)
Time to reach familiarization (seconds) ^1^	60 (53)	52 (21)	54 (31)
Average run duration (seconds) ^1^	4.6 (2.2)	4.4 (2.4)	4.3 (6.6)

Note: percentages may not sum to 100 due to rounding, medical record abstraction has not been completed for all CIOB participants, and some data currently noted as missing will be obtained through medical record abstraction. Abbreviations: SD, standard deviation. ^1^—Participants’ data were only included in mean calculations and data analysis if infants completed all trials of the VRM. If infants only completed a portion of the VRM task, their data were coded as incomplete. In IKIDS, 260 infants have VRM outcome data included. The VRM task was implemented in CIOB in 2016, and to date, 73 infants have VRM data available. Participants enrolled before 2016 did not complete the VRM.

**Table 3:** Unadjusted associations between demographic characteristics and birth outcomes in total ECHO.CA.IL cohort.

Demographics	Gestational Age (Weeks)			Birth Weight Z-Score		
	*n*	β	95% CI	*n*	β	95% CI
Maternal Age (years)						
<25	99	−0.21	(−0.63, 0.21)	91	−0.18	(−0.42, 0.06)
25–29	251	Ref	Ref	216	Ref	Ref
30–34	508	−0.02	(−0.3, 0.25)	458	0.15	(−0.01, 0.31)
≥35	365	0.07	(−0.22, 0.36)	358	0.12	(−0.04, 0.29)
Pre-pregnancy BMI (kg/m^2^)						
Underweight (<18.5 kg/m^2^)	35	−0.53	(−1.11, 0.06)	31	0.02	(−0.34, 0.37)
Normal (18.5–24.9 kg/m^2^)	624	Ref	Ref	578	Ref	Ref
Overweight (25–29.9 kg/m^2^)	274	−0.10	(−0.34, 0.15)	254	0.19	(0.05, 0.34)
Obese (≥30 kg/m^2^)	231	−0.46	(−0.72, −0.20)	204	0.33	(0.18, 0.49)
Maternal Education						
<College degree	307	−0.44	(−0.70, −0.19)	283	−0.09	(−0.24, 0.07)
College degree	371	Ref	Ref	341	Ref	Ref
Graduate degree	523	0.09	(−0.13, 0.32)	477	0	(−0.13, 0.14)
Race/Ethnicity						
White	722	Ref	Ref	647	Ref	Ref
Black	70	−0.77	(−1.21, −0.33)	64	−0.67	(−0.92, −0.43)
Asian/Pacific Islander	160	−0.14	(−0.44, 0.17)	153	−0.44	(−0.61, −0.27)
Hispanic	206	−0.41	(−0.69, −0.13)	202	−0.18	(−0.33, −0.03)
Multi-Racial/Other	57	0.25	(−0.23, 0.73)	50	−0.37	(−0.65, −0.10)
Infant Sex						
Male	582	Ref	Ref	547	Ref	Ref
Female	629	−0.01	(−0.21, 0.19)	576	−0.02	(−0.14, 0.09)
Parity						
1+ prior births	565	Ref	Ref	522	Ref	Ref
No prior births	650	−0.06	(−0.26, 0.13)	594	0.33	(0.22, 0.44)
Current Smoker						
No	1212	Ref	Ref	1212	Ref	Ref
Yes	11	0.50	(−0.57, 1.57)	11	−0.35	(−0.93, 0.24)
Marital Status						
Married or living together	1083	Ref	Ref	989	Ref	Ref
Single	86	−0.27	(−0.65, 0.10)	81	−0.48	(−0.70, −0.26)

Abbreviations: BMI, body mass index; CI, confidence interval; Ref, reference.

**Table 4:** Unadjusted associations between demographic characteristics and visual recognition memory (VRM) outcomes in total ECHO.CA.IL cohort.

Demographics	Novelty Preference (Proportion)			Time to Reach Familiarization (Seconds)			Average Run Duration (Seconds)		
	*n*	β	95% CI	*n*	β	95% CI	*n*	β	95% CI
Maternal Age (years)									
<25	18	0.24	(−3.24, 3.72)	18	2.41	(−13.61, 18.43)	18	−0.01	(−3.39, 3.38)
25–29	89	Ref	Ref	89	Ref	Ref	89	Ref	Ref
30–34	156	0.01	(−1.78, 1.79)	156	3.10	(−5.13, 11.34)	156	0.04	(−1.70, 1.78)
≥35	70	-0.03	(−2.18, 2.13)	70	2.12	(−7.78, 12.02)	70	0.43	(−1.67, 2.52)
Pre-pregnancy BMI (kg/m^2^)									
Underweight (<18.5 kg/m^2^)	8	0.75	(−4.08, 5.58)	8	4.50	(−13.66, 22.66)	8	1.47	(−3.34, 6.27)
Normal (18.5–24.9 kg/m^2^)	168	Ref	Ref	168	Ref	Ref	168	Ref	Ref
Overweight (25–29.9 kg/m^2^)	73	−1.2	(−3.07, 0.67)	73	2.09	(−4.95, 9.12)	73	−0.39	(−2.25, 1.47)
Obese (≥30 kg/m^2^)	69	−0.61	(−2.52, 1.3)	69	3.39	(−3.78, 10.57)	69	−0.44	(−2.34, 1.46)
Maternal Education									
<College degree	50	−0.85	(−3.11, 1.41)	50	1.57	(−8.82, 11.95)	50	0.77	(−1.44, 2.98)
College degree	118	Ref	Ref	118	Ref	Ref	118	Ref	Ref
Graduate degree	165	0.61	(−1.00, 2.23)	165	−6.25	(−13.67, 1.17)	165	0.10	(−1.48, 1.67)
Race/Ethnicity									
White	244	Ref	Ref	244	Ref	Ref	244	Ref	Ref
Black	15	−0.35	(−3.93, 3.23)	15	3.72	(−12.56, 20)	15	−0.49	(−3.95, 2.97)
Asian/Pacific Islander	31	−0.22	(−2.78, 2.35)	31	−4.88	(−16.55, 6.8)	31	−0.97	(−3.46, 1.51)
Hispanic	22	1.32	(−1.68, 4.32)	22	19.48	(5.85, 33.1)	22	−3.36	(−6.26, −0.47)
Multiracial/other	21	−0.59	(−3.65, 2.47)	21	−4.22	(−18.14, 9.7)	21	−0.71	(−3.66, 2.25)
Infant Sex									
Male	156	Ref	Ref	156	Ref	Ref	156	Ref	Ref
Female	176	0.42	(−1.06, 1.90)	176	0.16	(−6.64, 6.96)	176	−0.39	(−1.61, 0.83)
Parity									
1+ prior births	150	Ref	Ref	150	Ref	Ref	150	Ref	Ref
No prior births	177	1.06	(−0.44, 2.55)	177	−0.92	(−6.48, 4.63)	177	−0.51	(−1.96, 0.95)

Abbreviations: BMI, body mass index; CI, confidence interval; Ref, reference. Note: marital status and smoking status were not included due to small sample sizes.

## Data Availability

The sharing of anonymized data from this study is restricted due to ethical and legal restrictions. Data contains sensitive personal health information, which is protected under Health Insurance Portability and Accountability Act (HIPPA), thus making all data requests subject to Institutional Review Board (IRB) approval. Per University of California, San Francisco (UCSF) IRB, the data that support the findings of this study are restricted for transmission to those outside the primary investigative team. Data sharing with investigators outside the team requires IRB approval. Data requests may be submitted to the Program on Reproductive Health and the Environment (PRHE) by contacting Lynn Harvey at Lynn.Harvey@ucsf.edu. All requests for anonymized data will be reviewed by PRHE and then submitted to the UCSF IRB for approval.

## Supplementary Materials

The following are available online at https://www.mdpi.com/1660-4601/18/2/742/s1, Table S1. STROBE Statement—Checklist of items that should be included in reports of cohort studies. Table S2. Distribution of psychosocial stressors across demographic characteristics in ECHO.CA.IL. Table S3. Crude associations between demographic characteristics and gestational age stratified by cohort. Table S4. Crude associations between demographic characteristics and birth weight z-scores stratified by cohort. Table S5. Crude associations between demographic characteristics and term birth weight (grams) overall and stratified by cohort. Table S6.Distribution of PFAS (ng/mL) in material serum stratified by cohort. Table S7. Distribution of PFAS chemicals (ng/mL) in material serum within ECHO.CA.IL (*n* = 789) and NHANES females in 2013–2014. Table S8. Geometric mean (geometric standard deviation) of PFAS (ng/mL) levels across demographic characteristics in ECHO.CA.IL.
