# Supplementary material for: Associations of Maternal Stress, Prenatal Exposure to Per- and Polyfluoroalkyl Substances (PFAS), and Demographic Risk Factors with Birth Outcomes and Offspring Neurodevelopment: An Overview of the ECHO.CA.IL Prospective Birth Cohorts
Source: Int J Environ Res Public Health. 2021 Jan 16;18(2):742. doi: 10.3390/ijerph18020742 (PMC7830765; doi:10.3390/ijerph18020742)
Supplement: Supplementary file 1 [file ijerph-18-00742-s001.pdf]

International Journal of *Environmental Research and Public Health*

MDPI

Table S1. STROBE Statement—Checklist of items that should be included in reports of cohort studies.

STROBE Statement Checklist Items	Item No	Recommendation	Page No
Title and abstract	1	(a) Indicate the study's design with a commonly used term in the title or the abstract	1
		(b) Provide in the abstract an informative and balanced summary of what was done and what was found	1–2
		**Introduction**	
Background/ rationale	2	Explain the scientific background and rationale for the investigation being reported	2
Objectives	3	State specific objectives, including any prespecified hypotheses	3
		**Methods**	
Study design	4	Present key elements of study design early in the paper	3
Setting	5	Describe the setting, locations, and relevant dates, including periods of recruitment, exposure, follow-up, and data collection	3–4
Participants	6	(a) Give the eligibility criteria, and the sources and methods of selection of participants. Describe methods of follow-up	4
		(b) For matched studies, give matching criteria and number of exposed and unexposed	N/A
Variables	7	Clearly define all outcomes, exposures, predictors, potential confounders, and effect modifiers. Give diagnostic criteria, if applicable	4–6
Data sources/ measurement	8	For each variable of interest, give sources of data and details of methods of assessment (measurement). Describe comparability of assessment methods if there is more than one group	4–6
Bias	9	Describe any efforts to address potential sources of bias	7
Study size	10	Explain how the study size was arrived at	7
Quantitative variables	11	Explain how quantitative variables were handled in the analyses. If applicable, describe which groupings were chosen and why	5–6
Statistical methods	12	(a) Describe all statistical methods, including those used to control for confounding	7
		(b) Describe any methods used to examine subgroups and interactions	7
		(c) Explain how missing data were addressed	7
		(d) If applicable, explain how loss to follow-up was addressed	N/A
		(e) Describe any sensitivity analyses	7
		**Results**	
Participants	13	(a) Report numbers of individuals at each stage of study—eg numbers potentially eligible, examined for eligibility, confirmed eligible, included in the study, completing follow-up, and analysed	3-7
		(b) Give reasons for non-participation at each stage	N/A
		(c) Consider use of a flow diagram	N/A
Descriptive data	14	(a) Give characteristics of study participants (eg demographic, clinical, social) and information on exposures and potential confounders	7–9
		(b) Indicate number of participants with missing data for each variable of interest	7–9
		(c) Summarise follow-up time (eg, average and total amount)	3
Outcome data	15	Report numbers of outcome events or summary measures over time	8–9
Main results	16	(a) Give unadjusted estimates and, if applicable, confounder-adjusted estimates and their precision (eg, 95% confidence interval). Make clear which confounders were adjusted for and why they were included	8–11
		(b) Report category boundaries when continuous variables were categorized	5
		(c) If relevant, consider translating estimates of relative risk into absolute risk for a meaningful time period	N/A
Other analyses	17	Report other analyses done—eg analyses of subgroups and interactions, and sensitivity analyses	7
		**Discussion**	
Key results	18	Summarise key results with reference to study objectives	14
Limitations	19	Discuss limitations of the study, taking into account sources of potential bias or imprecision. Discuss both direction and magnitude of any potential bias	14–15
Interpretation	20	Give a cautious overall interpretation of results considering objectives, limitations, multiplicity of analyses, results from similar studies, and other relevant evidence	14
Generalisability	21	Discuss the generalisability (external validity) of the study results	15
		**Other information**	
Funding	22	Give the source of funding and the role of the funders for the present study and, if applicable, for the original study on which the present article is based	15

Note: An Explanation and Elaboration article discusses each checklist item and gives methodological background and published examples of transparent reporting. The STROBE checklist is best used in conjunction with this article (freely available on the Web sites of PLoS Medicine at http://www.plosmedicine.org/, Annals of Internal Medicine at http://www.annals.org/, and Epidemiology at http://www.epidem.com/). Information on the STROBE Initiative is available at http://www.strobe-statement.org.

Table S2. Distribution of psychosocial stressors across demographic characteristics in ECHO.CA.IL.

Demographics	Perceived Stress		Clinical Levels of Depression		Stressful Life Events	
	n	Mean (SD)	No *n* (%)	Yes *n* (%)	*n*	Mean (SD)
Maternal Age (years)						
<25	117	54.13 (10.79)	97 (8.1%)	15 (20.0%)	103	1.4 (1.21)
25–29	266	47.3 (10.93)	233 (19.6%)	22 (29.3%)	228	0.75 (1.07)
30–34	532	46.58 (9.42)	500 (42.0%)	21 (28.0%)	497	0.58 (0.86)
≥35	380	47.62 (9.2)	353 (29.6%)	14 (18.7%)	373	0.85 (1.05)
Pre-pregnancy BMI (kg/m²)						
Underweight (<18.5)	37	46.4 (7.87)	34 (2.9%)	1 (1.3%)	31	0.97 (0.91)
Normal (18.5–24.9)	629	46.82 (9.79)	588 (49.4%)	29 (38.7%)	588	0.63 (0.88)
Overweight (25–29.9)	282	48.2 (10.11)	252 (21.2%)	19 (25.3%)	262	0.88 (1.13)
Obese (≥30)	233	47.68 (10.42)	215 (18.1%)	15 (20.0%)	202	0.75 (1.02)
Education						
<College Degree	363	52.2 (10.47)	295 (24.8%)	50 (66.7%)	339	1.25 (1.21)
College Degree	363	45.9 (9.64)	361 (30.3%)	13 (17.3%)	339	0.69 (0.96)
Graduate Degree	536	45.93 (8.94)	518 (43.5%)	10 (13.3%)	499	0.49 (0.77)
Race/Ethnicity						
White	754	45.81 (9.96)	719 (60.4%)	23 (30.7%)	672	0.51 (0.81)
Black	73	53.1 (10.3)	63 (5.3%)	5 (6.7%)	66	1.35 (1.03)
Asian/Pacific Islander	167	48.54 (7.9)	149 (12.5%)	9 (12.0%)	160	0.89 (1.07)
Hispanic	246	51.71 (9.76)	201 (16.9%)	34 (45.3%)	254	1.25 (1.24)
Multi-Racial/Other	59	46.88 (9.98)	53 (4.5%)	2 (2.7%)	52	0.6 (0.87)
Infant Sex						
Male	595	47.75 (9.99)	549 (46.1%)	31 (41.3%)	571	0.73 (0.99)
Female	637	47.38 (9.94)	583 (49.0%)	36 (48.0%)	603	0.76 (1.02)
Parity						
1+ Prior Births	577	47.59 (9.25)	592 (49.7%)	47 (62.7%)	537	0.76 (0.97)
No Prior Births	653	47.32 (10.48)	542 (45.5%)	18 (24.0%)	594	0.72 (1)
Current Smoker						
No	1278	47.73 (9.98)	1179 (98.2%)	69 (92.0%)	1186	0.76 (1.02)
Yes	19	50.45 (11.89)	15 (1.3%)	4 (5.3%)	16	1.62 (1.2)
Marital Status						
Married or Living Together	1146	47.11 (9.9)	1067 (89.6%)	55 (73.3%)	1060	0.7 (0.97)
Single	104	54.19 (8.99)	78 (6.5%)	17 (22.7%)	94	1.57 (1.17)

Abbreviations: SD, standard deviation; BMI, body mass index. Note: Percentages for depression may not sum to 100 due to missing values. Perceived stress was harmonized by converting the PSS-4 in CIOB and PSS-10 in IKIDS to T-scores using the NIH toolbox. Depression was measured using the CES-D in CIOB and EPDS in IKIDS and was dichotomized using existing clinical cut points (≥16 for CES-D and ≥13 for EPDS). Stressful life events were summed to create a continuous measure (range 0-5).

Table S3. Crude associations between demographic characteristics and gestational age stratified by cohort.

Demographics	CIOB			IKIDS		
	n	β	95% CI	*n*	β	95% CI
Maternal Age (years)						
<25	56	0.06	(−0.62, 0.74)	43	−0.18	(−0.68, 0.32)
25–29	82	Ref	Ref	169	Ref	Ref
30–34	269	0.44	(−0.06, 0.93)	239	−0.24	(−0.53, 0.06)
≥35	289	0.64	(0.15, 1.13)	76	−0.49	(−0.9, −0.09)
Pre-pregnancy BMI (kg/m²)						
Underweight (<18.5 kg/m²)	23	−0.67	(−1.46, 0.13)	12	−0.26	(−1.12, 0.6)
Normal (18.5–24.9 kg/m²)	358	Ref	Ref	266	Ref	Ref
Overweight (25–29.9 kg/m²)	160	−0.17	(−0.52, 0.18)	114	0	(−0.32, 0.33)
Obese (≥30 kg/m²)	100	−0.55	(−0.96, −0.13)	131	−0.39	(−0.7, −0.08)
Maternal Education						
<College Degree	205	−0.67	(−1.04, −0.31)	102	−0.06	(−0.42, 0.31)
College Degree	182	Ref	Ref	189	Ref	Ref
Graduate Degree	287	0.12	(−0.22, 0.46)	236	0.04	(−0.24, 0.33)
Race/Ethnicity						
White	301	Ref	Ref	421	Ref	Ref

Black	41	−1.24	(−1.89, −0.6)	29	−0.2	(−0.76, 0.37)
Asian/Pacific Islander	130	−0.3	(−0.71, 0.11)	30	0.12	(−0.43, 0.68)
Hispanic	193	−0.57	(−0.93, −0.21)	13	0.16	(−0.67, 0.99)
Multi-Racial/Other	23	0.28	(−0.56, 1.12)	34	0.23	(−0.29, 0.76)
Infant Sex						
Male	328	Ref	Ref	254	Ref	Ref
Female	356	0.05	(−0.24, 0.34)	273	−0.09	(−0.35, 0.16)
Parity						
1+ Prior Births	357	Ref	Ref	208	Ref	Ref
No Prior Births	331	−0.13	(−0.42, 0.16)	319	0.01	(−0.25, 0.27)
Marital Status						
Married or Living Together	583	Ref	Ref	500	Ref	Ref
Single	59	−0.34	(−0.85, 0.16)	27	−0.11	(−0.69, 0.47)

Abbreviations: BMI, body mass index; CI, confidence interval; Ref, reference. Note: smoking status was not included due to a small sample size.

Table S4. Crude associations between demographic characteristics and birth weight z-scores stratified by cohort.

Demographics	CIOB			IKIDS		
	n	β	95% CI	*n*	β	95% CI
Maternal Age (years)						
<25	55	−0.18	(−0.53, 0.17)	36	−0.13	(−0.46, 0.21)
25–29	77	Ref	Ref	139	Ref	Ref
30–34	262	0.08	(−0.18, 0.33)	196	0.29	(0.09, 0.49)
≥35	288	0.16	(−0.09, 0.42)	70	0.17	(−0.1, 0.43)
Pre-pregnancy BMI (kg/m^2)						
Underweight (<18.5 kg/m^2)	22	−0.1	(−0.54, 0.34)	9	0.32	(−0.29, 0.93)
Normal (18.5–24.9 kg/m^2)	353	Ref	Ref	225	Ref	Ref
Overweight (25–29.9 kg/m^2)	157	0.14	(−0.06, 0.33)	97	0.29	(0.07, 0.51)
Obese (≥30 kg/m^2)	98	0.16	(−0.07, 0.39)	106	0.48	(0.27, 0.69)
Maternal Education						
<College Degree	198	−0.03	(−0.23, 0.17)	85	−0.12	(−0.37, 0.12)
College Degree	181	Ref	Ref	160	Ref	Ref
Graduate Degree	281	0.01	(−0.18, 0.2)	196	0.01	(−0.18, 0.2)
Race/Ethnicity						
White	294	Ref	Ref	353	Ref	Ref
Black	39	−0.64	(−0.98, −0.31)	25	−0.68	(−1.04, −0.31)
Asian/Pacific Islander	130	−0.33	(−0.53, −0.12)	23	−0.76	(−1.14, −0.38)
Hispanic	190	−0.14	(−0.32, 0.05)	12	0.12	(−0.4, 0.64)
Multi-Racial/Other	22	−0.14	(−0.57, 0.3)	28	−0.56	(−0.91, −0.21)
Infant Sex						
Male	326	Ref	Ref	221	Ref	Ref
Female	356	0.01	(−0.14, 0.16)	220	−0.06	(−0.23, 0.12)
Parity						
1+ Prior Births	351	Ref	Ref	171	Ref	Ref
No Prior Births	324	0.39	(0.24, 0.54)	270	0.2	(0.02, 0.38)
Marital Status						
Married or Living Together	571	Ref	Ref	418	Ref	Ref
Single	58	−0.39	(−0.66, −0.12)	23	−0.64	(−1.03, −0.25)

Abbreviations: BMI, body mass index; SD, standard deviation; Ref, reference. Note: smoking status was not included due to a small sample size.

Table S5. Crude associations between demographic characteristics and term birth weight (grams) overall and stratified by cohort.

Demographics	Total			CIOB			IKIDS		
	n	β	95% CI	*n*	β	95% CI	*n*	β	95% CI
Maternal Age (years)									
<25	84	−86.71	(−198.4, 24.98)	48	−79.78	(−244.29, 84.73)	36	−75.54	(−231.29, 80.21)
25–29	212	Ref	Ref	74	Ref	Ref	138	Ref	Ref
30–34	435	74.81	(2.25, 147.37)	245	51.11	(−66.64, 168.86)	190	124.67	(31.59, 217.75)
≥35	336	48.61	(−27.38, 124.59)	270	74.8	(−41.67, 191.28)	66	32.51	(−92.05, 157.06)
Pre-pregnancy BMI (kg/m^2)									

Underweight (<18.5 kg/m^2)	29	−35.62	(−201.77, 130.54)	20	−75.9	(−283.09, 131.3)	9	52.24	(−230.96, 335.44)
Normal (18.5–24.9 kg/m^2)	553	Ref	Ref	333	Ref	Ref	220	Ref	Ref
Overweight (25–29.9 kg/m^2)	240	75.07	(7.65, 142.48)	145	47.7	(−41.85, 137.24)	95	116.81	(14.57, 219.04)
Obese (≥30 kg/m^2)	195	100.41	(27.77, 173.05)	92	29.2	(−76.8, 135.2)	103	165.47	(66.05, 264.89)
Maternal Education									
<College Degree	264	−41.69	(−113.63, 30.25)	179	−34.54	(−130, 60.92)	85	−40.2	(−153.37, 72.97)
College Degree	326	Ref	Ref	169	Ref	Ref	157	Ref	Ref
Graduate Degree	461	14.05	(−48.83, 76.92)	273	6.81	(−80.3, 93.92)	188	30.59	(−60.27, 121.45)
Race/Ethnicity									
White	621	Ref	Ref	278	Ref	Ref	343	Ref	Ref
Black	58	−282.37	(−399.7, −165.04)	34	−269.74	(−429.63, −109.86)	24	−292.18	(−464.78, −119.59)
Asian/Pacific Islander	145	−185.22	(−264.04, −106.41)	122	−147.08	(−242.65, −51.51)	23	−327.65	(−503.72, −151.58)
Hispanic	186	−84.62	(−156.05, −13.19)	174	−82.19	(−167.25, 2.88)	12	62.52	(−177.54, 302.58)
Multi-Racial/Other	50	−140.7	(−266.32, −15.08)	22	−58.74	(−253.64, 136.16)	28	−205.42	(−366.08, −44.76)
Infant Sex									
Male	519	Ref	Ref	302	Ref	Ref	217	Ref	Ref
Female	539	−135.3	(−188.28, −82.33)	326	−122.62	(−193.3, −51.94)	213	−151.46	(−231.29, −71.62)
Parity									
1+ Prior Births	495	Ref	Ref	329	Ref	Ref	166	Ref	Ref
No Prior Births	566	132.33	(79.5, 185.16)	302	154.98	(85.35, 224.62)	264	86.41	(3.51, 169.31)
Marital Status									
Married or Living Together	945	Ref	Ref	538	Ref	Ref	407	Ref	Ref
Single	76	−234.25	(−337.13, −131.37)	53	−200.53	(−328.11, −72.94)	23	−291.29	(−469.41, −113.18)

Abbreviations: BMI, body mass index; SD, standard deviation; Ref, reference. Note: smoking status was not included due to a small sample size.

Table S6. Distribution of PFAS (ng/mL) in material serum stratified by cohort.

PFAS	CIOB (*n* = 510)						IKIDS (*n* = 279)					
	% Above MDL	% Machine Readable	Median	IQR	95% Percentile	Maximum	% Above MDL	% Machine Readable	Median	IQR	95% Percentile	Maximum
PFNA	98.82	99.61	0.3	(0.2, 0.44)	0.85	18.7	100	100	0.31	(0.20, 0.44)	0.77	2.28
PFOS	100	100	1.94	(1.19, 3.14)	6.04	14.5	99.64	99.64	2.45	(1.51, 3.99)	7.50	21.1
PFOA	99.8	100	0.76	(0.46, 1.12)	2.11	32.2	100	100	0.85	(0.46, 1.25)	2.43	4.85
Me-PFOSA-AcOH	98.82	99.8	0.05	(0.03, 0.08)	0.19	1.79	93.91	93.91	0.05	(0.02, 0.11)	0.31	3.87
PFHxS	100	100	0.33	(0.2, 0.59)	1.52	4.94	98.57	98.57	0.59	(0.31, 1.01)	2.62	10.3
PFDeA	69.35	92.34	0.16	(0.12, 0.24)	0.5	3.87	96.77	97.13	0.09	(0.05, 0.15)	0.28	1.60
PFUdA	72.3	95.28	0.14	(<MDL, 0.23)	0.4	1.26	93.55	93.91	0.05	(0.03, 0.10)	0.23	1.92
PFOSA	2.36	45.97	<MDL	(<MDL, <MDL)	0.08	0.11	46.59	46.59	<MDL	(<MDL, 0.02)	0.04	0.15
PFBS	0.79	54.42	<MDL	(<MDL, <MDL)	0.09	0.1	34.05	34.05	<MDL	(<MDL, 0.01)	0.02	0.24
PFHpA	11.98	67.98	<MDL	(<MDL, <MDL)	0.17	0.49	53.76	53.76	0.02	(<MDL, 0.04)	0.10	0.27
PFDoA	2.16	58.35	<MDL	(<MDL, <MDL)	0.42	0.48	43.01	43.37	<MDL	(<MDL, 0.06)	0.16	0.47
Et-PFOSA-AcOH	10.61	69.74	<MDL	(<MDL, <MDL)	0.05	0.09	20.43	20.43	<MDL	(<MDL, <MDL)	0.08	0.14

Abbreviations: per- and polyfluoroalkyl substances, PFAS; MDL, method detection limit; IQR, interquartile range. Note: % machine readable indicates the percent of PFAS concentrations in which a signal was obtained. Median, IQR, 95th Percentile, and Maximum values were calculated with PFAS concentrations below the MDL coded as missing.

Table S7. Distribution of PFAS chemicals (ng/mL) in material serum within ECHO.CA.IL (*n* = 789) and NHANES females in 2013−2014.

PFAS	ECHO.CA.IL						NHANES	
	% Above MDL	% Machine Readable	Median	IQR	95% Percentile	Maximum	Median (95% CI)	95th percentile (95% CI)
PFNA	99.24	99.75	0.3	(0.2, 0.44)	0.84	18.7	0.60 (0.50–0.70)	1.90 (1.70–2.10)
PFOS	99.87	99.87	2.11	(1.27, 3.46)	6.63	21.1	4.00 (3.60–4.60)	15.10 (13.90–17.30)
PFOA	99.87	100	0.78	(0.46, 1.17)	2.19	32.2	1.67 (1.47–1.87)	5.07 (4.07–6.70)
Me-PFOSA-AcOH	97.08	97.72	0.05	(0.03, 0.09)	0.24	3.87	<LOD	0.81 (0.59–0.97)
PFHxS	99.49	99.49	0.4	(0.23, 0.75)	2.04	10.3	1.00 (0.90–0.10)	2.94 (1.75–3.74)
PFDeA	79.06	94.04	0.13	(0.09, 0.2)	0.41	3.87	0.20 (0.10–0.20)	0.70 (0.50–0.90)
PFUdA	79.82	94.8	0.1	(0.06, 0.18)	0.36	1.92	<LOD	0.50 (0.40–0.60)

PFOSA	18.02	46.19	<MDL	(<MDL, <MDL)	0.05	0.15	<LOD	<LOD
PFBS	12.56	47.21	<MDL	(<MDL, <MDL)	0.03	0.24	<LOD	<LOD
PFHpA	26.78	62.94	<MDL	(<MDL, 0.07)	0.15	0.49	<LOD	0.20 (0.10–0.30)
PFDoA	16.62	53.05	<MDL	(<MDL, <MDL)	0.26	0.48	<LOD	0.20 (0.10–0.30)
Et-PFOSA-AcOH	14.09	52.28	<MDL	(<MDL, <MDL)	0.06	0.14	<LOD*	0.10 (<LOD–0.14)

Abbreviations: per- and polyfluoroalkyl substances, PFAS; MDL, method detection limit; LOD; limit of detection; IQR, interquartile range. Note: % machine readable indicates the percent of PFAS concentrations in which a signal was obtained. Median, IQR, 95th Percentile, and Maximum values were calculated with PFAS concentrations below the MDL coded as missing. Me-PFOSA-AcOH is called the following in NHANES: 2-(N-Methyl-perfluorooctane sulfonamido) acetic acid (MeFOSAA), PFDeA, perfluorodecanoic acid, is called the following in NHANES: PFDA, PFUdA, perfluoroundecanoic acid, is called the following in NHANES: PFUnDA, Et-PFOSA-AcOH is called the following in NHANES: 2-(N-Ethyl-perfluorooctane sulfonamido) acetic acid (EtFOSAA).

Table S8. Geometric mean (geometric standard deviation) of PFAS (ng/mL) levels across demographic characteristics in ECHO.CA.IL.

Demographics	PFNA			PFOS			PFOA			Me-PFOSA-AcOH			PFHxS			PFDeA			PFUdA		
	n	Mean (SD)	*p*	*n*	Mean (SD)	*p*	*n*	Mean (SD)	*p*	*n*	Mean (SD)	*p*	*n*	Mean (SD)	*p*	*n*	Mean (SD)	*p*	*n*	Mean (SD)	*p*
Maternal Age (years)																					
<25	78	0.25 (2.16)	0.78	77	1.64 (2.14)	0.48	78	0.62 (2.02)	0.13	76	0.06 (2.69)	0.26	78	0.37 (2.55)	0.38	68	0.09 (2.28)	0.84	72	0.04 (2.88)	0.53
25–29	163	0.26 (2.05)	Ref.	163	1.78 (2.92)	Ref.	163	0.71 (1.99)	Ref.	158	0.05 (3.32)	Ref.	161	0.41 (3.34)	Ref.	153	0.09 (2.16)	Ref.	153	0.05 (3.02)	Ref.
30–34	304	0.31 (2.04)	<0.01	305	2.24 (2.06)	<0.01	305	0.79 (2.1)	0.14	297	0.05 (2.95)	0.3	304	0.48 (2.54)	0.12	287	0.11 (2.48)	<0.01	287	0.08 (2.96)	<0.01
≥35	228	0.32 (1.76)	<0.01	229	2.03 (1.96)	0.11	229	0.75 (1.91)	0.50	226	0.05 (2.47)	0.92	228	0.36 (2.03)	0.21	221	0.13 (2.15)	<0.01	223	0.11 (2.51)	<0.01
Pre-pregnancy BMI (kg/m²)																					
Underweight (<18.5 kg/m²)	20	0.36 (2.11)	0.44	20	2.38 (2.46)	0.72	20	0.72 (2.07)	0.44	20	0.06 (3.33)	0.59	20	0.39 (2.72)	0.28	19	0.14 (3)	0.51	20	0.09 (3.28)	0.72
Normal (18.5–24.9 kg/m²)	388	0.32 (1.93)	Ref.	388	2.23 (2.22)	Ref.	388	0.81 (2.01)	Ref.	377	0.06 (2.72)	Ref.	386	0.49 (2.61)	Ref.	366	0.12 (2.29)	Ref.	371	0.09 (2.93)	Ref.
Overweight (25–29.9 kg/m²)	182	0.29 (2.16)	0.22	183	1.93 (2.25)	0.05	183	0.74 (2.07)	0.14	179	0.04 (2.95)	<0.01	183	0.39 (2.35)	<0.01	172	0.11 (2.41)	0.38	171	0.08 (2.96)	0.25
Obese (≥30 kg/m²)	153	0.24 (1.84)	<0.01	152	1.67 (2.21)	<0.01	153	0.61 (1.88)	<0.01	150	0.04 (2.98)	<0.01	151	0.32 (2.46)	<0.01	144	0.08 (2.09)	<0.01	143	0.04 (2.7)	<0.01
Maternal Education																					
<College Degree	242	0.23 (1.95)	<0.01	242	1.46 (2.17)	<0.01	24	0.57 (1.91)	<0.01	240	0.05 (2.52)	0.94	242	0.28 (2.34)	<0.01	217	0.09 (2.22)	<0.01	223	0.05 (2.68)	<0.01
College Degree	221	0.32 (1.96)	Ref.	222	2.15 (2.43)	Ref.	222	0.8 (2.11)	Ref.	215	0.05 (3.39)	Ref.	219	0.44 (2.89)	Ref.	213	0.11 (2.33)	Ref.	210	0.08 (3.14)	Ref.
Graduate Degree	314	0.34 (1.93)	0.34	314	2.49 (1.97)	0.03	314	0.87 (1.91)	0.15	306	0.05 (2.76)	0.74	314	0.55 (2.26)	<0.01	303	0.13 (2.29)	0.12	306	0.1 (2.85)	0.02
Race/Ethnicity																					
White	415	0.32 (1.9)	Ref.	416	2.38 (2.02)	Ref.	416	0.85 (2.06)	Ref.	403	0.05 (3.2)	Ref.	414	0.58 (2.39)	Ref.	401	0.1 (2.32)	Ref.	399	0.07 (3.08)	Ref.
Black	52	0.2 (1.89)	<0.01	51	1.55 (2.22)	<0.01	52	0.51 (1.86)	<0.01	51	0.08 (2.59)	0.01	52	0.25 (2.41)	<0.01	46	0.07 (2.12)	<0.01	49	0.06 (2.08)	0.59
Asian/Pacific Islander	101	0.37 (1.93)	0.08	101	2.43 (2.29)	0.81	101	0.77 (1.93)	0.17	101	0.05 (2.68)	0.58	101	0.33 (2.72)	<0.01	95	0.18 (2.11)	<0.01	99	0.15 (3.32)	<0.01
Hispanic	178	0.23 (1.85)	<0.01	179	1.38 (2.07)	<0.01	179	0.59 (1.81)	<0.01	178	0.04 (2.25)	0.12	179	0.25 (2.1)	<0.01	163	0.1 (2.24)	0.41	164	0.06 (2.33)	0.20
Multi-Racial/Other	30	0.33 (3.1)	0.78	30	1.77 (4.02)	0.04	30	0.93 (2.12)	0.50	27	0.04 (1.89)	0.52	28	0.51 (2.66)	0.44	27	0.17 (2.15)	<0.01	27	0.1 (2.49)	0.03

Infant Sex																					
Male	350	0.29 (1.96)	Ref.	350	2.02 (2.37)	Ref.	351	0.75 (2.06)	Ref.	341	0.05 (2.66)	Ref.	349	0.42 (2.52)	Ref.	331	0.11 (2.42)	Ref.	332	0.07 (3.03)	Ref.
Female	409	0.29 (1.99)	0.88	410	2 (2.13)	0.90	410	0.74 (2)	0.85	402	0.05 (3.02)	0.10	408	0.41 (2.62)	0.63	385	0.11 (2.26)	0.67	389	0.07 (2.96)	0.81
Parity																					
1+ Prior Births	353	0.36 (1.95)	Ref.	353	2.47 (2.24)	Ref.	353	1.05 (1.82)	Ref.	348	0.05 (2.73)	Ref.	352	0.59 (2.45)	Ref.	335	0.13 (2.31)	Ref.	342	0.09 (3)	Ref.
No Prior Births	423	0.25 (1.93)	<0.01	424	1.71 (2.15)	<0.01	425	0.56 (1.92)	<0.01	412	0.05 (2.97)	0.86	422	0.31 (2.42)	<0.01	398	0.09 (2.29)	<0.01	396	0.06 (2.89)	<0.01
Marital Status																					
Married or Living Together	705	0.3 (2)	Ref.	707	2.08 (2.25)	Ref.	707	0.76 (2.03)	Ref.	691	0.05 (2.9)	Ref.	703	0.44 (2.59)	Ref.	667	0.11 (2.34)	Ref.	670	0.08 (3)	Ref.
Single	71	0.24 (1.85)	0.01	70	1.56 (1.97)	<0.01	71	0.6 (1.86)	<0.01	69	0.06 (2.43)	0.13	71	0.29 (2.17)	<0.01	65	0.09 (1.99)	0.05	68	0.05 (2.63)	<0.01

Abbreviations: SD, standard deviation; Ref., reference. Note: Mean (SD) represents geometric mean (geometric standard deviation). Smoking status was not included due to a small sample size.
